# Advancements in Molecular Breeding Techniques for Soybeans

**DOI:** 10.3390/plants15010005

**Published:** 2025-12-19

**Authors:** Ivan Fetisov, Olga Eizikovich, Dominique Charles Diouf, Elena Romanova, Parfait Kezimana

**Affiliations:** Department of Agrobiotechnology, Peoples’ Friendship University of Russia (RUDN University), 117198 Moscow, Russia; fetisov-iv@pfur.ru (I.F.); eyzikovich_ov@pfur.ru (O.E.); dominiquediouf@hotmail.com (D.C.D.); romanova_ev@pfur.ru (E.R.)

**Keywords:** molecular breeding, soybean improvement, marker-assisted selection, genomic selection, CRISPR/Cas9, RNA interference, quantitative trait loci, stress tolerance, nutritional enhancement, crop improvement

## Abstract

Recent advances in molecular breeding techniques have greatly accelerated the development of improved soybean varieties with enhanced agronomic and nutritional traits. This review summarizes current research on innovative molecular approaches, including marker-assisted selection (MAS), genomic selection (GS), CRISPR/Cas9-mediated gene editing, and RNA interference (RNAi) for soybean improvement. Marker-assisted selection using simple sequence repeats (SSRs) and single-nucleotide polymorphisms (SNPs) has facilitated the efficient identification and incorporation of desired traits such as disease resistance, abiotic stress tolerance, and improved seed quality. Genomic selection has improved prediction accuracy for complex quantitative traits such as yield by integrating genome-wide molecular markers with phenotypic data. CRISPR/Cas9 technology has enabled precise genetic modification, resulting in soybeans with improved oil composition, increased isoflavone content and resistance to biotic stresses. RNA interference has successfully modulated gene expression to optimize nutritional properties and stress responses. These molecular breeding approaches overcome the limitations of traditional methods by shortening the breeding cycle and allowing for simultaneous improvement of multiple traits. The integration of these complementary techniques offers promising avenues for developing climate-resilient, high-yielding soybean varieties with improved nutritional profiles to address global food security challenges.

## 1. Introduction

Soybean (*Glycine max*), an annual herbaceous plant of the legume family (*Fabacea*), is one of the most common leguminous crops cultivated in many countries. It is a very valuable technical, fodder and food crop due to its high content of protein, fats, vitamins and amino acids. However, soybean production is under threat due to increased salinity, frequent droughts, floods, unusual temperature fluctuations, and the nature and frequency of precipitation. The negative effects of climate change are further exacerbating the situation.

With the growing global demand for high-yielding and resilient soybean varieties, traditional breeding methods have faced numerous limitations, leading to the need for innovative approaches such as molecular breeding. This technique uses molecular biology and genomic tools to accelerate the genetic improvement of soybean varieties, ultimately improving their yield, disease resistance and adaptability to environmental stresses [1].

Traditional breeding methods, while fundamental to the history of agriculture, have several limitations that limit their effectiveness in developing new soybean varieties. For example, classical breeding relies heavily on sexual reproduction between compatible plants, which limits the addition of novel traits that may not exist within the same species. In addition, these methods are often time-consuming, requiring multiple generations of breeding to achieve desirable traits. As a result, the limitations imposed by genetic diversity and long breeding cycles hinder the rapid production of high-performing soybean varieties that can meet the demands of modern agriculture [2].

In response to the limitations of traditional techniques, advances in molecular breeding techniques have ushered in a new era of crop improvement. Innovations such as marker-assisted selection (MAS) and genomic selection allow breeders to more efficiently identify and utilize specific genetic markers associated with desirable [3]. For example, CRISPR/Cas9 gene-editing technology has changed the breeding landscape by enabling precise changes to DNA sequences, enabling targeted improvements in traits such as oil content, seed size, and stress resistance [4]. These technologies hold great promise for both vegetable and grain soybeans, allowing both agronomic and quality traits to be incorporated into desired varieties more quickly than traditional methods.

The application of molecular breeding techniques has had a profound impact on the improvement of soybean varieties. In vegetable soybeans, these advances can improve traits such as taste, texture, and nutritional profile to better meet consumer demand [1]. Overall, the interplay of genomic technologies and molecular markers is revolutionizing the breeding process and promoting the development of superior soybean varieties that can thrive in diverse environments.

This review focuses on advances in molecular breeding techniques for soybean, the need for which is underscored by the rapid development of these methods and their critical impact on food security. A comprehensive review of current research provides insights into the integration of various omics approaches, genetics, and breeding techniques that are essential for understanding the complexities involved in soybean improvement. Moreover, this knowledge can guide future research efforts aimed at optimizing and tailoring genetic traits in vegetable soybean for specific end uses, ultimately enhancing both the agricultural and nutritional value of this vital crop.

## 2. Overview of Molecular Breeding Techniques

**Figure 1:**
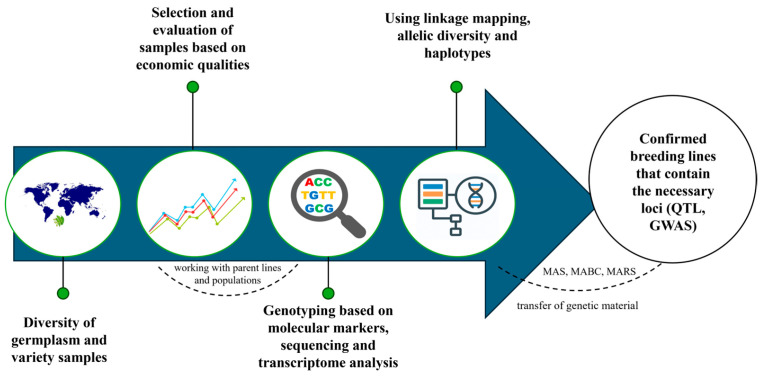
Integrated approaches to soybean genetics and breeding to improve nutritional qualities, adapted from [8].

Soybean (*Glycine max* L.) is a legume crop grown for food, feed, and industrial use, as well as for an alternative fuel production route [5]. It is used in crop rotations for more sustainable agricultural practices due to its nitrogen-fixing properties [6]. Soybean is economically important due to its role in meeting the growing demand for high-protein foods and oil, which increases its importance. Traditional soybean breeding methods, although successful in many areas, face several limitations [7]. There are difficulties with the genetic improvement of protein content, as there is a negative correlation with oil content, yield and resistance to temperature influences. To overcome these challenges, modern molecular breeding techniques have emerged as powerful tools. To improve the nutritional properties of soybeans, comprehensive approaches are needed, which include genetic mapping and the use of markers and loci of quantitative traits. Figure 1 shows the study and integration of various germplasm, followed by the study of the phenotype and genotype, the determination of the necessary QTL and further study in marker-oriented selection and genome-wide associations study to improve the nutritional qualities of soybeans [8].

Modern molecular breeding encompasses four major approaches: marker-assisted selection (MAS), genomic selection (GS), CRISPR/Cas9 gene editing, and RNA interference (RNAi). Each technique offers unique advantages for trait improvement. Marker-assisted selection (MAS) has become a tool for improving the efficiency and accuracy of breeding. MAS uses molecular markers associated with desired characteristics, which allows breeders to select plants at early stages of the selection process based on their genetic composition. This method reduces the time and resources needed to breed new varieties. Studies have demonstrated the effectiveness of quantitative trait loci (QTL) associated with key characteristics such as protein content in seeds and oil [9,10]. The combination of MAS with other modern technologies, such as genomic breeding and CRISPR/Cas9 editing, makes it possible to accelerate further research to improve the quality of soybeans and the breeding of new varieties [7]. Genomic selection (GS) differs from MAS by using more molecular markers to assess breeding value, the presence of candidate genes, and the prediction of certain characteristics without determining the marker–trait relationship. With the development of high-performance genotyping methods and the constant optimization of statistical models, the use of GS in breeding has become more effective.

CRISPR/Cas9-based gene editing is the simplest, most flexible, and most accurate method for plant genetic editing. It is based on Cas9 endonuclease and guide RNA (gRNA). gRNA consists of two small RNA molecules: CRISPR RNA (crRNA, the complementary DNA of the target) and transactivating crRNA (tracrRNA). Recognition of the target sequence using Cas9 requires the presence of a specific motif adjacent to the protospacer (PAM), directly adjacent to the target site. PAM requirements are one of the main limitations of the CRISPR/Cas9 system, as it reduces flexibility in selecting a target site [11].

RNA silencing or RNA interference (RNAi) refers to various RNA-mediated pathways for precisely inhibiting gene expression, both at the post-transcriptional and transcriptional levels. This provides a powerful tool for suppressing a gene or family of genes, allowing researchers to analyze their functions, including for duplicated genes.

Thus, modern methods of molecular breeding allow for more accurate and rapid breeding of new soybean lines with the desired characteristics. At the same time, they overcome some of the limitations of classical breeding and make it possible to improve even those traits that are in negative correlation with each other. In addition, the methods allow us to study the functionality of candidate genes and describe them, which also speeds up further breeding work.

## 3. Molecular Markers in Soybean Breeding

The use of molecular markers for soybean yield improvement represents a major advance in plant breeding. These tools offer greater precision and efficiency in identifying and selecting genes or genomic regions (QTL) associated with complex traits such as yield. Marker-based molecular analyses confirm the presence of genetic variability among the genotypes studied. Several studies have shown that molecular markers can help improve soybean genotypes and develop high-yielding varieties [12,13]. The use of molecular breeding is considered a viable method for improving resistance to certain soybean diseases and minimizing adverse effects [12]. Studies have identified genes for resistance to important soybean diseases such as brown stem rot, downy mildew, sclerotinia, etc. Molecular markers have become indispensable tools for improving soybean disease resistance. They provide greater precision and efficiency in identifying and selecting resistance genes. With these tools, researchers and breeders can develop healthier soybean varieties, helping to improve food security and reduce the environmental impact of agriculture [14]. The production of drought-tolerant soybean varieties is an important goal for many plant breeders, but progress to date has been slow. Intensive research efforts have identified many genes and processes affected by drought in soybean [15]. A set of robust phenotypic and molecular markers is needed to aid in the evaluation of cultivars for stress tolerance. The application of a potential marker in the field must also consider the existing infrastructure of the sites where the marker will be used and the technical expertise required for accurate evaluation. While some technologies, such as molecular markers or “omics” approaches, are excellent tools for laboratory analysis, they are technically demanding, often expensive and often require specific skills [16]. Stress tolerance is often controlled by multiple genes acting in synergy. Molecular markers facilitate the selection of plants that combine several tolerance genes that can have a cumulative effect on stress resistance. This approach makes it possible to develop more robust soybean varieties that can cope with a wider range of environmental stresses.

The use of molecular markers represents a significant advance in improving the nutritional quality of soybeans. These tools provide a precise and efficient approach to identifying and selecting the genes involved in seed composition, opening new prospects for developing more nutritious varieties adapted to consumer needs. The development of cutting-edge tools and technologies, including high-throughput genotyping, phenotyping, DNA sequencing techniques, and genome editing, has enabled great strides to be made in improving the functional and nutritional qualities of soybean [17].

Molecular marker technology has evolved through three distinct generations, each representing significant technological advancements in detection method, throughput, and cost-efficiency. This evolution from gel-based to sequence-based markers has dramatically increased the precision and scalability of breeding programs

First Generation: AFLP Markers—Representing the first generation of high-throughput markers—AFLP markers are a molecular biology technique that combines restriction enzyme digestion and PCR amplification to analyze variations in the length of DNA fragments. In simple terms, this means that DNA is cut into pieces and then some of these pieces are amplified for comparison between different individuals. These are markers that rapidly analyze the genome and are ideal for assessing genetic variation in germplasm [18]. AFLPs are dominant markers that allow for the study of multiple loci simultaneously and generate highly reproducible markers that are also considered to be locus specific within a species [19].

Second Generation: Simple Sequence Repeats (SSR Markers)—SSR genotyping uses simple sequence repeats (SSRs) as DNA markers. SSRs, also known as microsatellites, are a type of repetitive DNA sequence that is ubiquitous in most plant genomes. SSRs contain repeats of a sequence of motifs from 1 to 6 bp in length. Because of this structure, SSRs are frequently mutated, mainly by DNA polymerase errors involving the addition or subtraction of a repeat unit [20,21]. As a result, SSR sequences are highly polymorphic and can be easily used to detect allelic variation within populations. SSRs are present in both genetic and nongenetic regions and are sometimes transcribed, and can therefore be identified in expressed sequence tags (ESTs) and more commonly in nongenetic DNA sequences [20]. SSR genotyping involves the design of DNA-based primers to amplify SSR sequences from extracted genomic DNA, followed by amplification of the SSR region by polymerase chain reaction, and then visualization of the resulting DNA products, usually by gel electrophoresis [22]. Key improvements over AFLPs included (1) codominant inheritance allowing for heterozygote detection, (2) locus-specific amplification improving reproducibility, and (3) high information content per marker.

Third Generation: SNP Markers—Third-generation Single-Nucleotide Polymorphism (SNP) markers represent a paradigm shift from gel-based to sequence-based detection. SNP markers are variations in a single nucleotide base in the DNA sequence. In other words, a change in a single “letter” of the genetic code at a specific location in the genome. These variations are very common and can exist between different individuals or populations. SNPs are more common and occur at high frequencies in both coding and non-coding regions of the genome [23]. SNPs are increasingly becoming the markers of choice for genetic study and selection. Furthermore, SNPs are the most common source of variation in eukaryotic genomes and variant calling, and are more accurate due to their bi-allelic nature [24,25]. They are highly abundant and stable, making them valuable tools for breeding. They are easy to manipulate and analyze using modern molecular biology techniques. They can be used to identify genomic regions (QTL) associated with complex traits [26]. SNPs markers are particularly used to identify genes or QTL associated with yield. These studies help us to better understand the genetic factors controlling this complex trait and to select the most productive varieties [27]. The provided advantages over previous generations include (1) abundance (millions of potential markers), (2) automation (no gel reading), (3) bi-allelic nature (clear scoring), (4) genome-wide coverage, and (5) decreasing per-datapoint costs. SNPs have become the marker of choice for modern genomic selection and genome-wide association studies.

## 4. Marker-Assisted Breeding

The marker-assisted selection (MAS) method makes it possible to significantly speed up the breeding process by selecting target genotypes from a small sample of plants already at the initial stages of breeding. Soybean (*Glycine max*) has become an object for MAS, as many quantitative trait loci (QTL) have been mapped in the genome of this plant. They are defined. Important signs include grain quality, resistance to phytopathogens, tolerance to various abiotic factors, vitamin content, seed weight, and many other signs.

This technology is also used to study the genetic mechanisms of mineral accumulation in beans, which creates the basis for improving nutritional qualities (biofortification) and yield using MAS methods [12].

One of the qualitative features of soybeans is the oil content. Seeds contain a large amount of fatty acids (about 20% of the seed weight), the main ones are stearic acid, palmitic acid, linolenic acid and oleic acid [13]. In one study, 6 QTL were identified, which are responsible for the content of oleic acid. They are located on chromosomes 5, 17, 18, and 19 [28].

One of the achievements can be considered the development of a panel of markers for genes that control the soy photoperiod and growing season. Thus, the study tested various combinations of primers to detect different allelic states responsible for the desired traits of the genes. The main genes considered by the authors are the previously identified *E1*–*E4*, *GmFT5a* (*Glycine max* flowering time) and *GmFT2a*. The genes in question control flowering and maturation depending on daylight hours. It was also found that the precocity of varieties had a positive correlation with the number of recessive *E1*–*E4* genes [14].

MAS uses several types of molecular markers, each with its own advantages and applications. Simple repeating sequences (SSRS), also known as microsatellites. They are characterized by polymorphism and are widely used due to their high level of variability, codominant inheritance, and ease of detection [15]. When using MAS in breeding programs, molecular markers associated with traits of interest, such as yield, disease resistance, and stress tolerance, are first identified. Genetic mapping is performed and analysis with markers obtained during random DNA amplification (RAPD) is used. After receiving information about the necessary sites, genotyping of interbreeding populations is carried out to identify the presence of these markers. In the future, they often resort to backcrossing. The use of backcrossing with the participation of markers has received a separate name—MABC. This technology is common to obtain the necessary attributes in elite lines [7].

MAS was used to select soybean plants with resistance to early cracking of beans, which leads to large crop losses. For this study, the TaqMan SNP assay was successfully developed to detect the A/G allele of the KS-STP 5 marker in breeding materials. The overall accuracy of MAS prediction was 92.5% and 96.2% in two populations and breeding lines [16]. MAS was also used to produce vegetable soy lines that completely lacked a trypsin inhibitor, which is an anti-nutritional substance and spoils the economic qualities of the product. For this purpose, SSR markers were used, which made it possible to identify homozygous lines [17].

## 5. Genomic Selection

Genomic breeding (GS) is a new approach to improving quantitative traits in plant breeding populations that uses genome-wide molecular markers, namely high-density markers and high-throughput genotyping. Genomic forecasting combines data on a huge number of markers with genealogical and phenotypic data to increase the accuracy of predicting genetic value and is suitable for identifying complex quantitative traits (for example, yield) [29].

When using GS in crop breeding, the density of markers, the sample size, the relationship between the population underlying the statistical model and the population under test, the population structure, the heritability and genetic architecture of target traits, as well as the linkage disequilibrium (LD) between markers and QTL are considered [30].

In soybean breeding, researchers studied the effect of population structure on the accuracy of predicting yield increases. According to the results of the study, it became known that GS is applicable for this trait [31]. Other researchers conducted a genome-wide association search (GWAS) together with GS to study amino acid concentrations in soybean seeds. The results of the study showed that the efficiency of selection for markers that were precisely associated with amino acids was higher than when using random markers [28]. GS was used to study the effect on the oil and protein content in soybeans, considering the population size, phenotypic and genotypic similarity between the population underlying the statistical model and the estimated population, the number of markers and the marker-gene relationship. Research results have shown that GS is a potentially effective method of increasing the protein and oil content in soybeans [30].

In another example, GS was performed using a regression model of the best prediction, which shows the greatest effectiveness in evaluating loci that control complex features. The authors identified significant loci that controlled two or more traits. The region of chromosome 2 identified by the SNP markers Chr02_12086588 and Chr02_19239630 was associated with both seed weight and plant height. The 9-megabyte region of chromosome 4 containing SNPsChr04_46043483, Chr04_46043518, and Chr04_36949349 was significantly related to the maturation period and seed weight. The genomic sequence covering the region of chromosome 7 contained SNPs Chr07_33588669 and Chr07_7610107 and was associated with seed weight and yield, respectively. In addition, b on chromosome 8, identified by significant SNP markers Chr08_47483065 and Chr08_47747059, was associated with seed weight and yield [31].

Thus, genomic selection significantly speeds up the breeding process and significantly increases its efficiency [29].

## 6. CRISPR/Cas9 Technology

While marker-assisted selection and genomic selection enhance breeding efficiency by identifying favorable genetic variants, they are limited to variation existing within available germplasm. CRISPR/Cas9 overcomes this constraint by enabling direct, precise modification of target genes. This capability has proven particularly valuable in soybean, where the technology has been successfully applied to improve traits ranging from herbicide resistance to nutritional quality.

Soybean genome editing using the CRISPR/Cas9 system is classified into five types and two classes according to the characteristics of the Cas protein. In particular, a type II system based on the Cas9 protein from the bacterium Streptococcus pyogenes, which has been modified to improve the efficiency of genome editing, is used for genetic engineering. This system is relatively simple in structure and consists of three main components: the target site located directly in front of the protospacer-adjacent motif (PAM), the guide RNA (sgRNA) and the Cas9 protein [32]. The process of gene editing takes place in three stages: recognition, cutting and repair. sgRNA is an oligonucleotide with a length of 20–22 nucleotides, which makes it easy to construct a guide sequence for targeting Cas9 to any DNA with the appropriate motif (for example, 5′-N(20–22)-NGG-3′). The guide RNA contains a complementary sequence at the 5′ end, providing specific recognition of the target gene. Without sgRNA, Cas9 is unable to cut the target DNA. The Cas9 enzyme creates a double-stranded break in DNA about three nucleotides up from the PAM motif. After the rupture is formed, the cells repair it in two ways. The first pathway, non—homologous end joining (NHEJ), often leads to minor sequence changes, which can lead to gene knockout and loss of its function. The second pathway, homologous recombination reduction (HDR), allows for the insertion of a pre-prepared DNA template into the desired location of the genome [32].

Soybeans are also the only legume crop for which CRISPR technology has become widespread. In addition to editing, CRISPR/Cas9 is also used to study the functions of genes. A CRISPR library containing more than 100 candidate genes was created [11]. The researchers studied HD-Zip transcription factors, which play an important role in plant secondary metabolism and response to abiotic stress, about which little was known in soybeans. Using CRISPR/Cas9, plants with an edited target gene were created, and during the analyses, results were obtained that showed increased drought resistance of soybeans compared to controls and plants with increased gene expression [33]. This technology improved the oil and protein content, controlled the flowering time and architecture of plants, changed the content of isoflavones, and created resistant lines to various phytopathogens, such as soybean mosaic virus [11].

As an example, studies on *ASL* (Argininosuccinate lyase) genes highlight the use of this technology in soybean improvement. Argininosuccinate lyase (ASL) is a critical enzyme in the arginine biosynthesis pathway that catalyzes the reversible cleavage of arginosuccinate into arginine and fumarate. It plays multiple essential roles including nitrogen assimilation, amino acid biosynthesis, and metabolic regulation. The ASL gene family in soybean consists of four members (*ASL1*, *ASL2*, *ASL3*, and *ASL4*) located on chromosome 4. These genes are particularly important in herbicide metabolism pathways, where specific amino acid substitutions can confer resistance to herbicidal compounds without compromising plant fitness. In one allele, the *ASL1* codon was changed (from CCC to AGC), after which proline was changed to serine. Thus, the soybean plant was able to acquire resistance to herbicides [34].

Also, using CRISPR technology, scientists were able to make a knockout of the *GmFT2* gene responsible for the flowering of soybeans. After that, *GmFT2a* mutants were able to show delayed flowering time in both long and short daylight conditions [35].

In other studies, CRISPR/Cas9 technology helped researchers identify targets in experiments with root pubescence [36].

Researchers were also able to improve the nutritional properties of soybeans without affecting other agronomically valuable characteristics of the plant. Using CRISPR/Cas9, mutations were induced in two *GmGOLS1A* (*Glycine max* galactinol synthase) and *GmGOLS1B* genes encoding *GOLS* to study the role of these genes in RFO metabolism. *GOLS* synthase is responsible for the production of galactanol, which is a controlling factor in the biosynthesis of oligosaccharides of the raffinose family (RFOs), including raffinose, stachyose, cicerolite and verbascose. They cannot be broken down in the intestines of humans and animals due to the lack of the necessary enzyme (alpha-galactosidase) and accumulate in the large intestine. The result of the experiment was an improvement in the nutritional properties of soybeans due to a significant decrease in the level of RFOs in seeds (with single and double mutant knockout) [37].

One of the important qualitative indicators is the content of isoflavonoids. And the content of these substances in soybeans is about a hundred times higher than the content in other legumes. They are important both for maintaining human health and for the “health” of the plant itself, namely, they significantly increase resistance to various phytopathogens. The synthesis of isoflavonoid compounds is regulated by enzymes such as flavone synthase (*IFS*), flavanone-3-hydroxylase (*F3H*) and flavone synthase II (*FNS II*). Several loci have been modified using the CRISPR/Cas9 technology in the hairy roots of soybeans and other plants. The genes involved in the synthesis of isoflavones, such as *GmF3H1* (*Glycine max* flavanone 3-hydroxylase), *GmF3H2*, as well as the genetic material of the T0 transgenic line, demonstrated a triple mutation that was stably transmitted to the next generation. It has been shown that the concentration of isoflavones in these leaves is significantly higher than in wild-type leaves. The content of isoflavones in the leaves of the triple mutant of the T3 zygote generation was twice as high as in wild soybeans. In addition, these leaves demonstrated immunity to the soy mosaic strain (SMV), which opens up new prospects for obtaining plants with an altered genome with potentially valuable agronomic qualities [38].

A few other examples are provided in Table 1.

## 7. RNA Interference

Complementing gene editing approaches, RNA interference (RNAi) offers an alternative strategy for trait improvement through sequence-specific gene silencing. Unlike CRISPR/Cas9, which creates permanent DNA modifications, RNAi modulates gene expression at the post-transcriptional level without altering the genome sequence. RNAi is a natural mechanism for regulating gene expression found in plants, which provides protection against viruses and controls the activity of mobile genetic elements [52]. This endogenous pathway has been successfully harnessed in soybean for both functional genomics research and crop improvement. The process includes the following key steps:

1. Formation of double RNA (dsRNA): dsRNA can occur in a cell as a result of viral infection, transposon activity, or artificial injection. Plant viruses are able to induce the synthesis of double-stranded RNA, which triggers the protective mechanisms of RNAi [53,54,55].
2. dsRNA processing: The Dicer enzyme cleaves dsRNA into short interfering RNAs (siRNAs), about 21–24 nucleotides long. These small RNAs play a central role in post-transcriptional silencing of genes [56].
3. Formation of the RISC complex: siRNAs integrate into the RISC complex (RNA-induced silencing complex), which uses them as a template for searching for complementary mRNAs [57].
4. Degradation or blockade of mRNA: After binding to the target mRNA, RISC initiates its cleavage or prevents translation, which leads to a decrease in the expression of the corresponding gene [52].

Due to these mechanisms, RNAi plays an important role in the immune defense of plants and is widely used in biotechnology to create crops with improved characteristics, including resistance to viruses and changes in the expression of desired genes [56].

**Figure 2:**
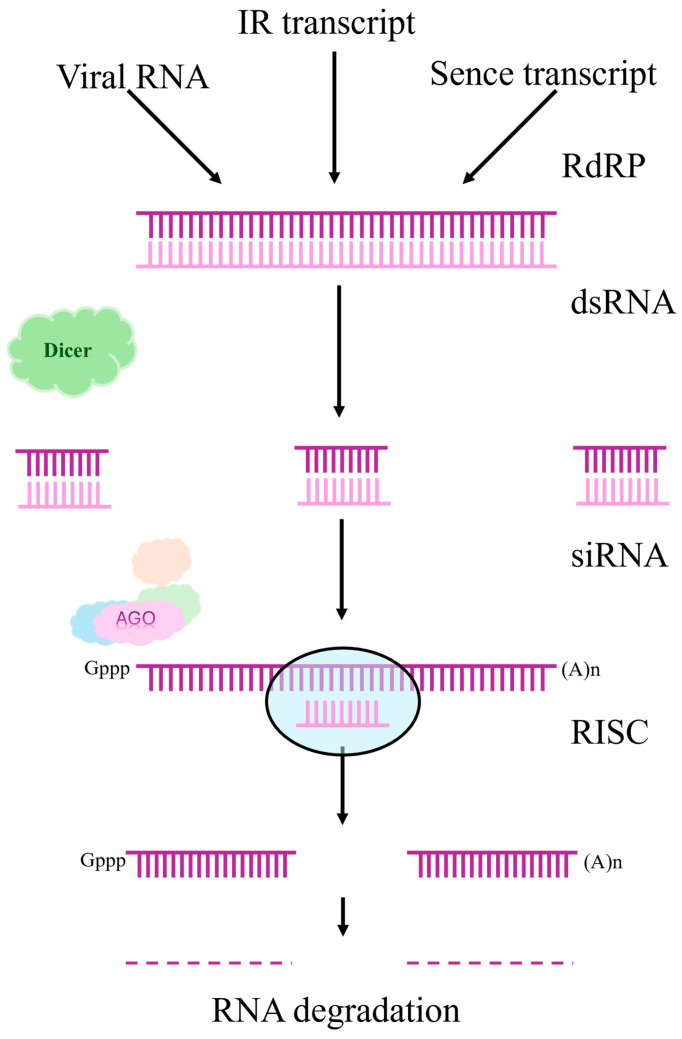
Pathways of silencing, adapted from [58].

RNA silencing can be either caused by researchers or nature [58]. Figure 2 shows the silencing pathways used to suppress the regulation of the target gene through RNA degradation:

For soybean transgenic lines, a transgene was often used that transcribes semantic RNA homologous to the gene to silence the necessary sequences—co-suppression. But the more common method is using an inverted repeat (IR) of the target gene, which forms double-stranded RNAs (dsRNAs) during transcription (IR-PTGS), which later degrades. Mechanisms based on viral vectors, which underlie the protective reaction of plants, are also used. Thus, a viral vector was used as a source for the induction of a silencing-specific gene in the plant genome, which is called virus-induced gene silencing (VIGS). Currently, three vectors are available for soybeans: based on bean pod mottle virus (BPMV), cucumber mosaic virus (CMV) and apple latent spherical virus (ALSV) [58]. RNA interference has been used by researchers to enhance the synthesis of isoflavones in soy, by synthesizing and introducing a divalent RNAi vector [59].

In one study, the authors, using RNAi, were able to prove that when suppressing the expression of *IFS1* (*Glycine max* isoflavone synthase 1) and *IFS2*, soybean plants become more susceptible to Phytophthora sojae. Moreover, stability was impaired not only in the roots, but also non-specific stability in cotyledons [60].

There are also studies on the nitrogen-fixing ability of soybeans. The authors found that suppression of *GmS6K1* (S6 Kinase 1) expression by RNA interference disrupted nodule development, significantly reducing their quality and halving nitrogen fixation [61].

In another study, the authors used RNAi to increase isoflavone production. They created a bivalent vector for transformation based on RNAi, which suppressed the expression of two genes (*F3H* and *GmFNSII* (*Glycine max* flavone synthase II)). Reducing the expression of these genes using RNA interference effectively controlled the synthesis of flavones and isoflavones in the “hairy” roots formed from soybean cotyledons transformed by *Agrobacterium rhizogenes* ATCC15834. It turned out that the bivalent RNAi vector had a significantly more pronounced effect on increasing isoflavone synthesis compared with the use of two separate RNAi vectors. To determine the effect of expression suppression, the authors analyzed the roots of independent positive transformations. As a result, significant differences in the levels of both individual and total isoflavones were recorded. Thus, the authors were able to conclude that divalent RNAi roots proved to be significantly more effective for increasing isoflavone production than two-element RNAi roots [59].

## 8. Conclusions

The use of advanced molecular breeding of soybeans can significantly improve and accelerate the breeding process. The integration of advanced molecular breeding techniques represents a paradigm shift in soybean improvement strategies, offering unprecedented precision and efficiency compared to conventional breeding approaches. This review has shown how molecular markers, genomic selection, CRISPR/Cas9-mediated gene editing, and RNA interference have collectively transformed soybean breeding by enabling rapid and targeted genetic improvement.

Marker-assisted selection using SSR and SNP markers has proven particularly effective in identifying and incorporating disease resistance genes, quality traits and stress tolerance factors, while reducing the time required for phenotypic evaluation. Genomic selection extends these capabilities by enabling the prediction of complex quantitative traits through comprehensive genome-wide analysis, thereby increasing the accuracy of selection for yield and compositional traits. The precision of CRISPR/Cas9 technology has enabled targeted modification of genes regulating key metabolic pathways, resulting in soybeans with improved nutritional profiles, altered flowering time and enhanced pathogen resistance. Complementing these approaches, RNAi has provided a valuable tool for modulating gene expression and elucidating gene function in nitrogen fixation and isoflavone biosynthesis.

The complementary application of these molecular techniques addresses several critical limitations of traditional breeding, including long development cycles, limited genetic diversity, and challenges in selecting for multiple traits simultaneously. Future advances are likely to involve further integration of these complementary approaches with emerging technologies such as artificial intelligence and machine learning to optimize breeding decisions.

As global agricultural systems face increasing challenges from climate change and population growth, these molecular breeding strategies provide essential tools for developing high-yielding, nutritionally improved and environmentally resilient soybean varieties. Continued research and investment in these technologies, along with appropriate regulatory frameworks, will be critical to translating these scientific advances into tangible benefits for agricultural sustainability and global food security.

## Figures and Tables

**Table 1:** Application of CRISPR/Cas9 technology with a *Glycine max*.

Application	Target Gene	Mode of Action/ Method	Improved Trait	Reference
Increase crop yield (Regulating architecture and flowering period)	*SPL9a*, *SPL9b*	Knockout	Influence features of plant architecture	[39]
	*SPL9c*, *SPL9d*	Knockout	Influence features of plant architecture	[39]
	*GmLHY1a*, *b*,	Knockout	Influence features of plant architecture	[40]
	*GmLHY2a*, *2b*,	Knockout	Influence features of plant architecture	[40]
	*GmFT2a*, *GmFT4*	Base editing	Affect time of flower	[41]
	*GmAP1*	Knockout	Affect time of flower	[41]
	*Gmprr37*	Knockout	Affect time of flower	[42]
	*GmE1*	Knockout	Affect time of flower	[43]
	*GmFT2a*	Knockout	Affect time of flower	[44]
Enhance crop quality & nutrition (Storage protein/seed oil/sugar content/allergenic properties/bean flavor free Soybean)	*Glyma.03g163500*	Knockout	Regulate storage of protein	[45]
	*Glyma.20g148400*	Knockout	Regulate storage of protein	[45]
	*Glyma.19g164900*	Knockout	Regulate storage of protein	[45]
	*FAD2-2*	Knockout	Improve content of fatty acid	[46]
	*GmFAD2-1A*	Knockout	Improve content of fatty acid	[47]
	*GmFAD2-1B*	Knockout	Improve content of fatty acid	[47]
	*GmFAD2-1A*	Knockout	Improve content of fatty acid	[48]
	*GmFAD2-2A*	Knockout	Improve content of fatty acid	[48]
	*Glyma.19G147300*	Knockout	Improve content of fatty acid	[48]
	*GmFATB1* (*GmFATB1a* and *GmFATB1b*)	Knockout	Develop fatty acid content	[49]
	*GmGOLS1A*	Knockout	Develop fatty acid content	[49]
	*GmGOLS1B*	Knockout	Develop fatty acid content	[37]
	*GmSWEET15a*	Knockout	Develop fatty acid content	[50]
	*Gly m Bd 30K*	Knockout	Regulate allergenic	[51]

## Data Availability

No new data were created or analyzed in this study.
